# Active Trimming of High‐Speed SiN Photonic Micro Ring Resonators Using Thin Film Ferroelectrics

**DOI:** 10.1002/nap2.70059

**Published:** 2026-04-04

**Authors:** Tom Vanmaele, Enes Lievens, Kobe De Geest, Ewout Picavet, Andreas Laemont, Dries Van Thourhout, Peter Bienstman, Jeroen Beeckman

**Affiliations:** ^1^ Department of Electronics and Information Systems Ghent University Gent Belgium; ^2^ Photonics Research Group, Department of Information Technology Ghent University‐imec Gent Belgium; ^3^ SCRiPTS, Department of Chemistry Ghent University Ghent Belgium

**Keywords:** electro‐optic effect, ferroelectric materials, integrated photonics, post‐fabrication trimming

## Abstract

Fabrication of photonic devices needs nanometer‐scale precision for devices to be in the optimal point of operation. Achieving this precision typically requires complex and costly post‐fabrication processes or thermo‐optic tuning using resistive heaters. In this work, we demonstrate active trimming of ring resonators on a SiN platform using a ${\text{Pb}\left(\text{Zr,Ti}\right)O}_{3}$ cladding layer. A DC bias is applied in order to tune an optical modulator in the optimal point of operation while maintaining a constant high‐frequency response. This approach is promising for high‐speed applications where precise tuning is critical.

## Graphical abstract

This work successfully shows an active trimming technique for high‐speed optical devices. The trimming is performed on existing high‐speed architecture without altering its high‐speed capabilities. This leads to novel ways for increasing the yield in integrated photonics.

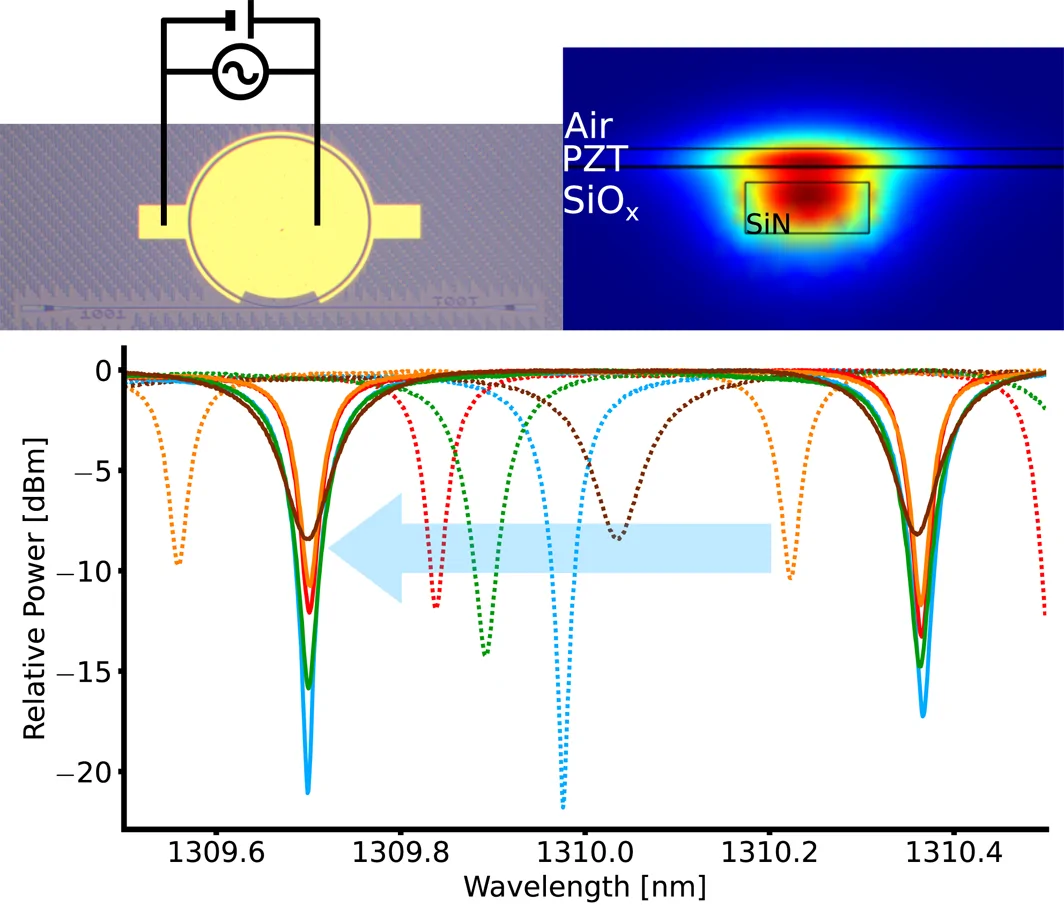

## 1 Introduction

Optical communication has become the backbone of the telecommunication industry over the last decades. The field of integrated silicon photonics is growing exponentially in order to keep up with the growth of the optical data stream. Silicon photonics has allowed for the rapid growth of integrated photonics because of its compatibility with CMOS foundries. Although it has seen great success, it might not be the ideal platform for integrated photonics. Indeed, active components require the co‐integration of novel materials such as III‐V materials for lasers and detectors and ferroelectric materials for high speed modulators [1, 2]. Integrated photonic components mostly work in analog operation, causing them to have much higher precision constraints than integrated electronics. These stringent precision requirements combined with the challenges of material integration cause serious issues in the yield of integrated photonic devices [3].

High‐speed optical modulators are a crucial component for encoding data onto an optical signal. They often operate by changing the refractive index of a material which in turn induces a phase change in the light. This phase change can then be used to directly encode data. It can also be used in an interferometer in order to achieve different levels of intensity [4, 5]. Commercially available integrated modulators use the plasma dispersion effect in silicon in order to induce a phase change. However, this also introduces spurious intensity modulation and is limited in speed by carrier mobility [4]. In order to achieve higher modulation speeds without intensity modulation, the Pockels effect in ferroelectric materials such as ${\text{LiNbO}}_{3}$ (LN), ${\text{BaTiO}}_{3}$ (BTO), and ${\text{Pb}\left(\text{Zr,Ti}\right)O}_{3}$ (PZT) have been used [4, 6, 7, 8]. In a SiN platform, the integration of these materials is especially crucial because it cannot use the plasma dispersion effect. Major issues for up‐scaling these modulators are fabrication errors which could cause unforeseen changes in the effective refractive index. When the refractive index is only slightly different, the wavelength at which the interference structure operates will shift.

Several ways exist to mitigate these errors using post‐fabrication trimming. Here, the devices can receive post‐fabrication treatment with a laser, integrated heaters, or liquid crystals to permanently alter the composition of the waveguide [9, 10, 11, 12, 13, 14]. This would require extra fabrication steps, where the device has to be meticulously monitored in order to ensure the changes are done in the correct way. This adds complexity to the fabrication process. The trimming can be done actively as well, where the device is integrated with heaters or active layers [15, 16]. This requires extra fabrication steps as well as increased power consumption as these heaters or active layers require constant power to operate.

In this work, we demonstrate an active trimming technique applied directly during high‐speed modulation, thereby eliminating the need for additional fabrication steps. Our experimental platform consists of five identical SiN ring resonators integrated with PZT, a ferroelectric material analogous to LN and BTO. PZT offers a distinct advantage in enabling simultaneous DC and AC operation due to its high remanent polarization. Once poled with a strong DC electric field, PZT exhibits a stable electro‐optic response across a wide DC bias range. This behavior contrasts with BTO, where the relatively low remanent polarization leads to a significant variation in the electro‐optic effect under changing DC conditions [6]. Furthermore, unlike LN, PZT does not suffer from DC drift—a phenomenon in which the electro‐optic effect induced by a DC field degrades over time [17]. The absence of DC drift in PZT makes it particularly suitable for a reliable and long‐term dual‐mode operation. The field of 2D ferroelectrics is rising within the photonic community because of their low power consumption, fast ferroelectric polarization switching, and other benefits. 2D ferroelectrics can be integrated heterogeneously. However, production at large scale is still difficult [18, 19]. The PZT films proposed in this work are solution‐deposited and epitaxially grown. This can be done on a large scale without increasing the cost or fabrication time significantly, providing an advantage over 2D materials in terms of scale.

## 2 Operating Mechanism

Both the refractive index and the high‐speed electro‐optic (Pockels) effect in a ferroelectric crystal depend on the orientation of the polarization vector $\vec{p}$ relative to the incident light. The following section introduces how this orientation influences the Pockels effect and refractive index in a polycrystalline tetragonal (P4mm) PZT film. The corresponding unit cell is shown in Figure 1a, and the system geometry is illustrated in Figure 1b. The polycrystalline PZT layer is epitaxially grown on an intermediate seed layer [8], resulting in random in‐plane crystal axes and a fixed out‐of‐plane crystal axis. For simplicity, an out‐of‐plane polarization angle of θ=0 is assumed. An in‐depth derivation can be found in Supporting Information S1 (S1 and S2).

**FIGURE 1:**
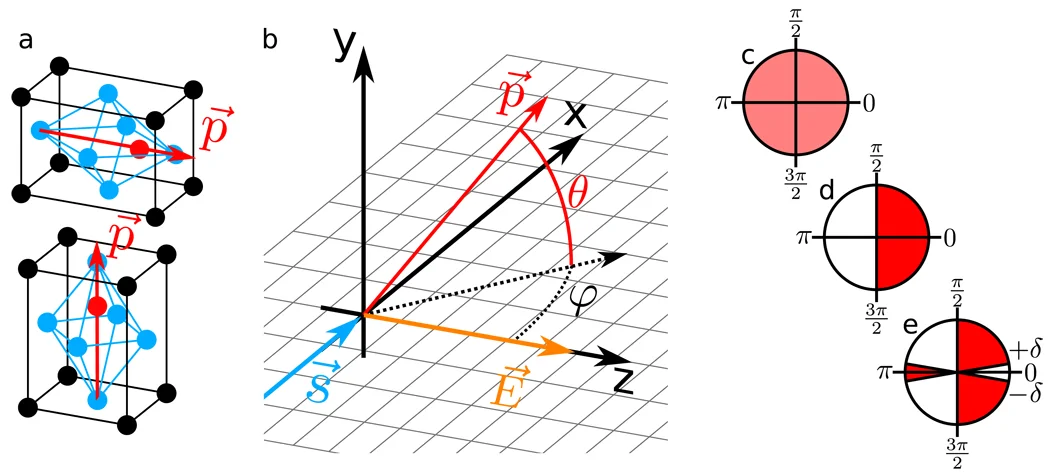
A ferroelectric crystal unit cell with the corresponding polarization vector $\vec{p}$ (a). Orientation of the polarization vector of the unit cell with respect to the propagation vector of light $\vec{s}$ and the applied electric field vector $\vec{E}$ (b). The distribution of the in‐plane rotation angle φ of a polycrystalline tetragonal ferroelectric material for a virgin sample (c), fully poled sample (d) and limited amount of domain reversal (e).

### 2.1 Electro‐Optic Effect

The orientation‐ and electric‐field‐dependent refractive index for TE polarization can be expressed as

(1) $$\begin{array}{c} n_{\text{eff}}=\frac{1}{n_{o}^{2}}+\left(\frac{1}{n_{e}^{2}}-\frac{1}{n_{o}^{2}}\right)\cos^{2}\;\varphi \\ +E\;\cos\;\varphi \left[r_{13}+2r_{51}+\cos^{2}\;\varphi \left(r_{33}-r_{13}-2r_{51}\right)\right] \end{array}$$

This relation shows how the effective refractive index $n_{\text{eff}}$ varies with the in‐plane crystal orientation φ and the applied electric field E. The ordinary and extraordinary refractive indices $\left(n_{o},n_{e}\right)$ and the electro‐optic coefficients $\left(r_{33},r_{13},r_{51}\right)$ are material constants describing how the field modifies the refractive index through the Pockels effect.

### 2.2 Ferroelectric Switching

Grain switching plays an important role in polycrystalline ferroelectric thin films. Although the 90° domain switching has been observed in PZT [20], this study only considers 180° switching. This simplifies the model while still being able to capture the effects observed during the experiments. The initial distribution of polarization angles φ is illustrated in Figure 1c. When a large E‐field is applied, the grains undergo a 180° switch to better align with the applied field, as shown in Figure 1d. The effective refractive index in that case becomes

(2) $$n_{\text{eff}}\approx n_{\mathrm{avg}}-\frac{En_{\mathrm{avg}}^{3}}{3\pi }\left(2r_{33}+2r_{51}+r_{13}\right)$$

where $n_{\mathrm{avg}}=\sqrt{2}n_{e}n_{o}/\sqrt{n_{o}^{2}+n_{e}^{2}}$. If the structure is fully poled in the opposite direction, the sign of the electric field term is reversed.

When the E‐field is lowered, some domains may switch back due to depolarization fields [21]. Grains most susceptible to domain reversal are those for which the influence of the net depolarization field is strongest, typically at φ=0 as shown in Figure 1e. Since a 180° switch does not affect the constant refractive index term, $n_{\mathrm{avg}}$ remains unchanged. The new effective refractive index is

(3) $$\begin{array}{c} n_{\text{eff}}=n_{\mathrm{avg}}-\frac{En_{\mathrm{avg}}^{3}}{3\pi }\left(2r_{33}+2r_{51}+r_{13}\right)\\ +2\;\sin\;\delta \frac{En_{\mathrm{avg}}^{3}}{3\pi }\left[3r_{33}+\left(2r_{51}+r_{13}-r_{33}\right)\sin^{2}\;\delta \right] \end{array}$$

Three distinct contribution are seen in Equation (3). The first is the electric field‐independent term, $n_{\mathrm{avg}}$, representing the zeroth‐order effective refractive index. The second is the linear, field‐dependent term corresponding to the first‐order electro‐optic response. The third term introduces a correction due to partial domain reversal. Together, these describe the effective refractive index of a polycrystalline tetragonal ferroelectric thin film in a regime where the sample is fully poled and limited domain reversal occurs. This regime applies when a strong electric field is applied and then reduced while remaining above the coercive field. In this region, the zeroth‐order refractive index is constant and the first‐order correction is small, enabling linear trimming of the device without degrading high‐speed electro‐optic performance—the central objective of this work.

## 3 Passive Response of Ring Resonators

In order to characterize and validate trimming operation, five identical ring resonators were fabricated in a photonic foundry and PZT was deposited in‐house. No special steps were taken to either increase or decrease the standard errors present in the manufacturing. The rings were placed in proximity to each other in order to emulate real‐live operation and variations. An image of one such ring is given in Figure 2a. The integrated stack with the propagating mode can be seen in Figure 2b. The actual transmission spectra of the rings after deposition of PZT are shown in Figure 2c. The most important part of this transmission spectrum is the position and shape of the resonance peaks. The free spectral range (FSR) and the spread on the resonance wavelengths of the ring resonators are 0.6 and 0.39 nm, respectively. There is also a spread on the extinction ratio (ER) and full width half maximum (FWHM) of the rings. This indicates in difference in losses or coupling between the waveguide and the ring. During trimming, a phase shift of more than π will be needed to compensate for the fabrication errors observed in the rings. Accomplishing a 2π would be ideal, since in that case any error can be compensated. In what follows, we will analyze the active response of these rings to characterize their behavior under an applied electric field. The final goal is to compensate the fabrication errors in these devices.

**FIGURE 2:**
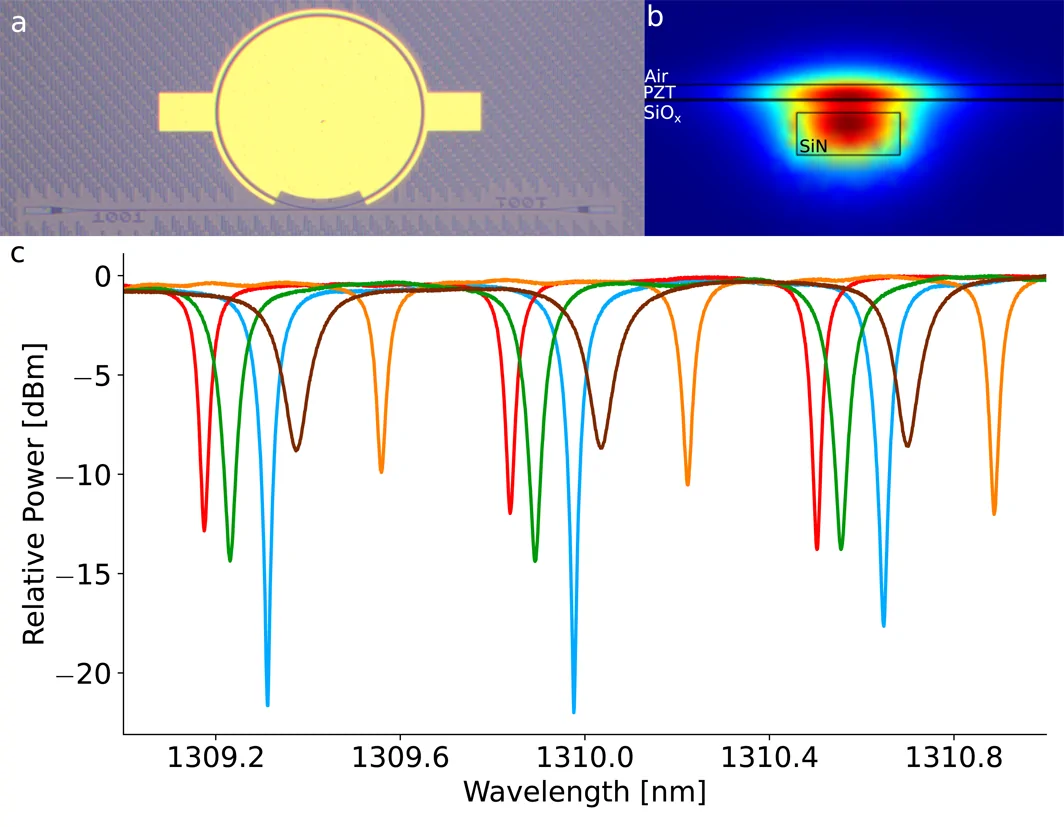
Microscope image of one of five identical ring resonators (a). Simulated intensity profile of the effective mode inside the integrated stack (b). Transmission spectra of five identical ring resonators in SiN, with PZT layer on top (c).

## 4 Active Response Under Applied Electric Fields

Multiple experiments were conducted to characterize and control the electro‐optic behavior of the devices. First, the ferroelectric thin film's hysteresis was measured. Next, its stability was evaluated to probe for DC drift. The third test examined high‐speed stability over a wide DC bias range, and finally, fabrication errors were compensated using a constant DC bias.

### 4.1 Ferroelectric Hysteresis

Hysteresis measurements were performed in two regimes: constant signal and small signal. The electric field was incrementally varied in steps of 0.333 V μm^−1^, with each electric field level maintained for 60 s. Following this period, the corresponding resonance wavelength was recorded. Subsequently, a small‐signal modulation was applied around each constant electric field level, ranging from ± 0.5 V μm^−1^ in steps of 0.25 V μm^−1^. At each step of this small‐signal variation, the resonance wavelength was again recorded. These measurements were then used to calculate the small‐signal tuning efficiency at each constant electric field. For the hysteresis characterization, the electric field is varied between ± 8.333 V μm^−1^. The resulting constant and small‐signal responses are given by Figure 3a,b.

**FIGURE 3:**
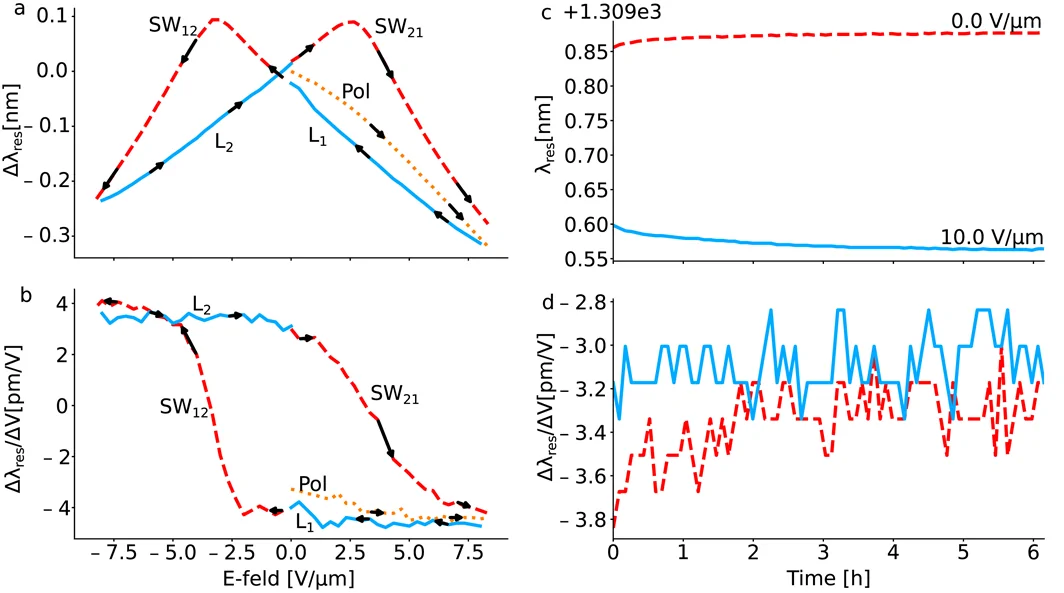
The resonance wavelength shift (a) and tuning efficiency (b) for a ring resonator at different E‐fields. The arrows on the graph show in what order the electric fields were applied. The resonance wavelength shift (c) and tuning efficiency (d) over time for two E‐fields.

There are five different regions on these graphs. The first is the poling region (Pol), where the device is poled from an initial state. The grains in the ferroelectric material switch from their initial state to align with the applied electric field. Upon lowering the electric field, the device enters the first linear region $\left(L_{1}\right)$. In this region, there is very little to no domain reversal in the grains. This results in a linear variation of the resonance wavelength and a constant tuning efficiency. When the electric field is lowered even further, the device enters the switching region $\left({\text{SW}}_{12}\right)$. Here, the grains switch until the device is fully poled in the opposite direction of $L_{1}$. When the electric field is increased again, the device enters the second linear region $\left(L_{2}\right)$. This is reflected in an opposite sign of tuning efficiency, which is expected from the theory. Finally, when the field keeps increasing, a second switching region $\left({\text{SW}}_{21}\right)$ occurs, where the device switches from $L_{2}$ to $L_{1}$.

It is within these linear regions that the device is operated. These regions will allow us to tune the devices over a broad DC‐range while still maintaining a constant high‐speed response. The slope remains linear well below or above 0 V μm^−1^ for positive or negative poling, respectively. This means that once the material is poled, it can be operated in the same regime even if the field is removed. This results in a very forgiving system in case of failure. In addition, it allows for low power operation where the device only needs to be tuned when it is operated and can be idle the rest of the time.

The ferroelectric switching might suggest a permanent change in the refractive index. This has not been observed when operating within the linear regions since here no polarization switching occurs. However, when operated within the switching region, a permanent change can be observed and has been shown in other research [22]. Whether this effect can be observed on this SiN‐PZT platform is under further investigation, as well as the operating principle of this phenomenon.

### 4.2 DC‐Drift

The DC drift was investigated by tracking the resonance wavelength while applying different electric fields over a 6‐h period. At every 5 min interval, a constant and small‐signal measurement was performed. The results are shown in Figure 3c,d. The resonance wavelength stays virtually unchanged over the 6‐h period. There is a slight change in the beginning, which can be accounted for by a small amount of grains which still switch, but compared to the overall shift, this change is negligible. This cannot be attributed to the same DC‐drift phenomenon observed in LN, where the effect is much larger [17]. This causes tuning of PZT with constant DC‐fields to be a reliable method for error correction in integrated optical devices. The tuning efficiency remains constant over time as well. Showing high‐speed operation remains unaltered over time.

### 4.3 High‐Speed Stability

The high‐speed stability is verified by first poling the device with a large electric field of 13.333 V μm^−1^ and then lowering the field in steps of 0.833 V μm^−1^ while continuously monitoring the high‐speed response. This was done to verify the concurrent operation between the high‐speed response and the applied DC biases. The high‐speed response is described as the magnitude of intensity modulation induced by the incoming RF signal at a specific wavelength. This response is quantified by the S12 parameter. The intensity modulation is optimal on the slope of a resonance peak in the spectrum. Since we are changing the applied electric field, the resonance wavelengths change as well. For each step, the wavelength at which the high‐speed response was measured was changed accordingly. The results for the ring resonators are shown in Figure 4. It can be seen that the 3‐dB cut‐off is between 10 and 15 GHz. This cut‐off is mainly governed by the design of the rings. Ring resonators are not ideal for high‐speed operation due to their inherently low bandwidth, which is limited by the photon lifetime within the ring [23]. The photon lifetime is defined as the time a photon spends on average in the ring and thus cannot contribute to the signal. The bandwidth is therefore limited to BW=1/2πτ, where τ is the photon lifetime. The photon lifetime is also related to the FWHM through $\Delta \omega_{\mathrm{FWHM}}=1/\tau$ [23]. The final calculation of the bandwidth of a ring resonator is given by

(4) $$BW=\frac{c\Delta \lambda_{\mathrm{FWHM}}}{\lambda^{2}}$$

**FIGURE 4:**
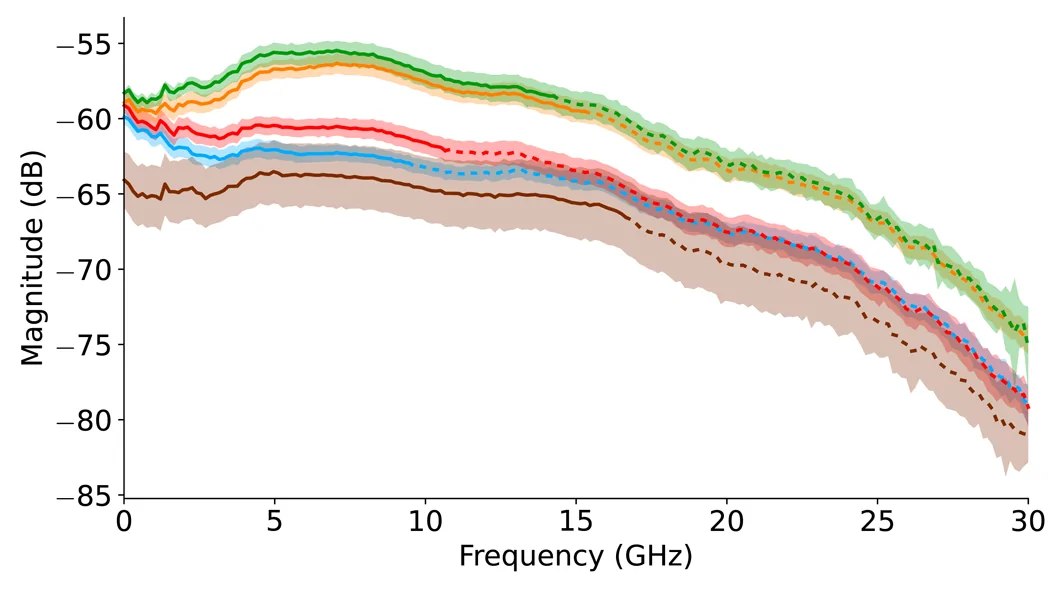
S12 parameters of five identical ring resonators, with a DC bias from 3.33 to 11.166 V μm^−1^. The 3‐dB bandwidth is indicated by the dashed line.

The calculated bandwidths of the rings are 13, 9.7, 9.3, 14.7, and 18.9 GHz, which is in line with what we observe in the S12 parameters. This shows us that the limiting factor for high‐speed modulation is the design of the component rather than the material. Even with this low cut‐off frequency, it can be seen that the applied bias field has no influence on the high‐speed response. The variation on the high‐speed response between different devices is also minimal, showing that fabrication errors mainly play a role in the position of the resonance wavelength rather than the high‐speed response.

### 4.4 Error Elimination

In the final experiment, we validate our active trimming approach by compensating fabrication‐induced resonance wavelength deviations in SiN ring resonators. The application of DC electric fields ranging from 3.33 to 11.166 V μm^−1^ enabled a precise alignment of the resonance wavelengths across five nominally identical devices, as shown in Figure 5. The electric fields applied are 25, 29, 35, 50, and 78 V for the resonance given from the smallest to largest wavelength shift, respectively, which gives an approximate tuning efficiency of 8, 5.5, 6.7, 6.6, and 6.7 pm V^−1^. The values are not in line with what was seen earlier in Figure 3; however, the rings in this measurement start from an unpoled state, which skews an initial resonance wavelength. The applied electric fields fall well within the previously established regime of high‐speed stability. This ensures that trimming does not compromise RF performance. The target resonance wavelength was set to 1309.7 nm. Due to the linear electro‐optic response of poled PZT, each device could be individually tuned by adjusting the applied field. All resonance peaks were successfully shifted toward shorter wavelengths, demonstrating the method's capability to correct for fabrication errors. Continuous upward shifts in wavelength are not feasible due to the reversal of the Pockels effect upon re‐poling. In Figure 5, one of the resonance wavelengths is shifted over more than 50% of the FSR. The devices were not close to breakdown voltages, and the theory and results do not suggest the behavior to deviate if higher voltages are applied. This means that the resonance peaks can be shifted over the full FSR and by extend, the full spectrum trough appropriate voltage scaling.

**FIGURE 5:**
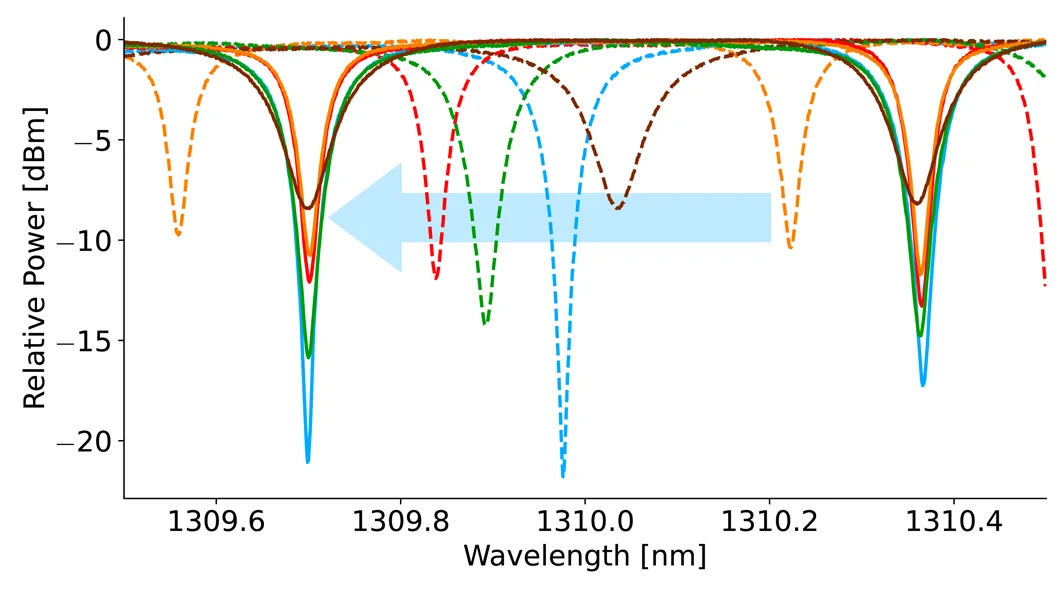
Transmission spectrum of five identical ring resonators, before and after trimming. The arrow indicates the trimming direction.

The ER and FWHM of the rings still remain different after tuning. The ER and FWHM are determined by the critical coupling of the ring resonators: the better the losses are matched to the coupling, the higher the ER and smaller the FWHM will be. If the ER and FWHM need to be adjusted, the coupling section of the ring resonator could be modulated as well.

## 5 Conclusion

This work demonstrates a robust and scalable method for the active trimming of high‐speed SiN photonic devices using thin‐film PZT ferroelectrics. By leveraging the stable electro‐optic properties of PZT, we successfully compensated for fabrication‐induced variations in resonance wavelength without compromising the RF performance of the device. The absence of DC drift in PZT, in contrast to materials like LN, further strengthens its candidacy for a long‐term, reliable operation in integrated photonic systems. Moreover, the trimming technique presented here is inherently compatible with existing high‐speed modulation architectures, requiring no additional fabrication steps or redesigns. Although the current study focused on ring resonators, the principles and mechanisms demonstrated are broadly applicable to other wavelength‐sensitive photonic components, such as Mach–Zehnder interferometers and filters. Future work could explore the integration of dynamic coupling control to further optimize Q‐factors post‐trimming, as well as investigate the long‐term reliability and environmental stability of PZT under varying operational conditions.

In summary, this study validates PZT as a high‐performance material for active trimming in SiN photonics. As the demand for precision and scalability in integrated optics continues to grow, techniques like the one presented here will be essential in bridging the gap between fabrication tolerances and functional performance.

## Author Contributions

**Tom Vanmaele:** conceptualization, investigation, writing – original draft, methodology, visualization, software. **Enes Lievens:** conceptualization, investigation, writing – review and editing. **Kobe De Geest:** software, visualization, writing – review and editing. **Ewout Picavet:** writing – review and editing. **Andreas Leamont:** writing – review and editing. **Dries Van Thourhout:** writing – review and editing. **Peter Bienstman:** writing – review and editing, supervision, funding acquisition. **Jeroen Beeckman:** writing – review and editing, supervision, funding acquisition, validation.

## Funding

This work was financially supported by Horizon Europe Project VISSION (Grant 101070622).

## Conflicts of Interest

The authors declare no conflicts of interest.

## Supporting information


Supporting Information S1

## Data Availability

All data, code, and materials used here are available to any researcher upon request.
