# Supplementary material for: Active Trimming of High‐Speed SiN Photonic Micro Ring Resonators Using Thin Film Ferroelectrics
Source: Nanophotonics. 2026 Apr 4;15(7):e70059. doi: 10.1002/nap2.70059 (PMC13642161; doi:10.1002/nap2.70059)
Supplement: Supplementary file 1 [file NAP2-15-e70059-s001.pdf]

Active Trimming of High-Speed SiN Photonic Micro Ring Resonators Using Thin Film Ferroelectrics: Supporting Information

1 Electro-optic effect

The orientation- and electric-field-dependent refractive index of light in birefringent materials can be derived using the index ellipsoid. We start from the constitutive equation:

$$\vec{E} = \frac{1}{\varepsilon_0}\bar{\bar{\eta}}\vec{D} \tag{1}$$

Combined with Maxwell's equations this gives [1]:

$$\vec{s} \times (\vec{s} \times \bar{\bar{\eta}} \cdot \vec{D}) = -\frac{1}{n^2}\vec{D} \tag{2}$$

The vector $\vec{s}$ represents the propagation vector of the light, in the coordinate system of the crystal. This can be rewritten to something resembling an eigenvalue problem:

$$(\bar{\bar{\delta}} - \vec{s} \times \vec{s}) \cdot \bar{\bar{\eta}} \cdot \vec{D} = \frac{1}{n^2}\vec{D} \tag{3}$$

Solving this eigenvalue problem will result in the 2 non-zero eigenvalues and 2 eigenvectors perpendicular to the propagation direction. In order to solve this problem, the orientation of the crystal needs to be defined. First an extrinsic rotation around the x-axis (1,0,0) over an angle θ defines the out-of-plane rotation. A second in-plane rotation is defined around the y-axis (0,1,0) over an angle φ. The impermeability and Pockels tensor are defined as $\bar{\bar{\eta}}$ and $\bar{\bar{\bar{r}}}$. The electric field vector is given by $\vec{\varepsilon}$. The Kerr effect is outside the scope of this derivation and because it is assumed to be negligible in this system.

$$\bar{\bar{\eta}} = \bar{\bar{\eta}}^{(0)} + \bar{\bar{\bar{r}}}\vec{\varepsilon} \tag{4}$$

In order to simplify the problem the electric field and Pockels coefficients are assumed to be small enough such that they do not introduce a significant change on the impermeability tensor, and we can apply perturbation theory. Instead of calculating the eigenvalues of the whole system at once, they are first calculated without an electric field applied. Then a small perturbation imposed by the Pockels effect on this solution is calculated, thus splitting the problem into two eigenvalue problems.
The polycrystalline material is epitaxially grown on an intermediate seed layer [2]. This results in a random in plane polarization but a fixed out-of plane polarization. We will assume an angle $\theta = 0$. This assumption is valid when only tetragonal phase of the material is considered, including rhombohedral phase would result in a positive or negative out of plane angle.

$$\eta_1 = \frac{1}{n_o^2} + E\cos\varphi r_{13} \tag{5}$$

$$\eta_2 = \frac{1}{n_o^2} + (\frac{1}{n_e^2} - \frac{1}{n_o^2})\cos^2\varphi + E\cos\varphi\left[r_{13} + 2r_{51} + \cos^2\varphi\,(r_{33} - r_{13} - 2r_{51})\right] \tag{6}$$

Because of the design of the components, mainly how light is coupled in using grating couplers, we are only interested in the TE polarization of the light[3]. Moving forward we can thus define one effective refractive index:

$$n_{eff} = \left(\frac{1}{n_o^2} + (\frac{1}{n_e^2} - \frac{1}{n_o^2})\cos^2\varphi + E\cos\varphi\left[r_{13} + 2r_{51} + \cos^2\varphi\,(r_{33} - r_{13} - 2r_{51})\right]\right)^{-\frac{1}{2}} \tag{7}$$

2 Ferroelectric switching

We now consider the switching behavior of a polycrystalline ferroelectric thin film. To maintain physical validity while keeping the model tractable, we restrict our analysis to 180° switching. In the thin film, the polarization of the grains is randomly oriented in-plane and aligned out-of-plane at angle $\theta = 0$. To determine the average refractive index of the structure, we first integrate the effective impermeability η_{eff} and then compute the inverse square root, i.e., $n_{eff} = 1/\sqrt{\eta_{eff}}$. In a poled sample, the grains have switched over 180° to align better with the applied electric field. The angles span uniformly from $-\frac{\pi}{2}$ to $\frac{\pi}{2}$. The effective refractive index in this case is given by:

$$\begin{aligned} n_{eff} &= \left(\frac{1}{\pi} \int_{-\frac{\pi}{2}}^{\frac{\pi}{2}} \frac{1}{n_o^2} + (\frac{1}{n_e^2} - \frac{1}{n_o^2}) \cos^2 \varphi + E \cos \varphi \left[r_{13} + 2r_{51} + \cos^2 \varphi \left(r_{33} - r_{13} - 2r_{51} \right) \right] d\varphi \right)^{-\frac{1}{2}} \\ &= \left(\frac{1}{n_{avg}^2} + E \frac{2}{3\pi} (2r_{33} + 2r_{51} + r_{13}) \right)^{-\frac{1}{2}} \\ &\approx n_{avg} - \frac{E}{3\pi} n_{avg}^3 (2r_{33} + 2r_{51} + r_{13}) \end{aligned} \tag{8}$$

where $n_{avg} = \sqrt{2} n_e n_o / \sqrt{n_o^2 + n_e^2}$. The above expression assumes a fully poled sample. We now consider a scenario in which the sample was initially fully poled, and the applied electric field is subsequently reduced. Under these conditions, some domains may switch back due to depolarization fields [4]. Grains most susceptible to domain reversal are those for which the influence of the net depolarization field is strongest, typically at $\varphi = 0$. We model this domain reversal as a small error δ in the span of the integral. Since a 180° switch does not affect the constant refractive index term, n_{avg} remains unchanged. The modified effective refractive index is then:

$$\begin{aligned} \eta'_{eff} = \eta_{eff} &- \frac{2}{\pi} \int_{\delta}^{\frac{\pi}{2}} E \cos \varphi \left[r_{13} + 2r_{51} + \cos^2 \varphi \left(r_{33} - r_{13} - 2r_{51} \right) \right] d\varphi \\ &+ \frac{1}{\pi} \int_{\pi-\delta}^{\pi+\delta} E \cos \varphi \left[r_{13} + 2r_{51} + \cos^2 \varphi \left(r_{33} - r_{13} - 2r_{51} \right) \right] d\varphi \end{aligned} \tag{9}$$

giving an n_{eff} of:

$$n_{eff} = \left(\frac{1}{n_{avg}^2} + E \frac{2}{3\pi} (2r_{33} + 2r_{51} + r_{13}) - 2 \sin \delta \left[3r_{33} + (2r_{51} + r_{13} - r_{33}) \sin^2 \delta \right] \right)^{-\frac{1}{2}} \tag{10}$$

The modified effective refractive index is then:

$$n_{eff} = n_{avg} - \frac{E n_{avg}^3}{3\pi} (2r_{33} + 2r_{51} + r_{13}) + 2 \sin \delta \frac{E n_{avg}^3}{3\pi} \left(\left[3r_{33} + (2r_{51} + r_{13} - r_{33}) \sin^2 \delta \right] \right) \tag{11}$$

3 Device parameters

The parameters of the ring resonators is given in table 1

4 Waveguide geometry simulation

The difference on the effective refractive index of the waveguide stack depending on a difference in waveguide width and hight is given in figure 1

The effective refractive index was simulated for the propagating mode with ±2 nm variation on the width and ±1 nm on the thickness of the waveguide. From these simulated refractive indices, we can roughly

Table 1: Ring resonator specifications

Radius	200 µm
Waveguide width	850 nm
Waveguide thickness	350 nm
Electrode spacing	6 µm
PZT thickness	120 nm
Waveguide and ring resonator gap	520 nm

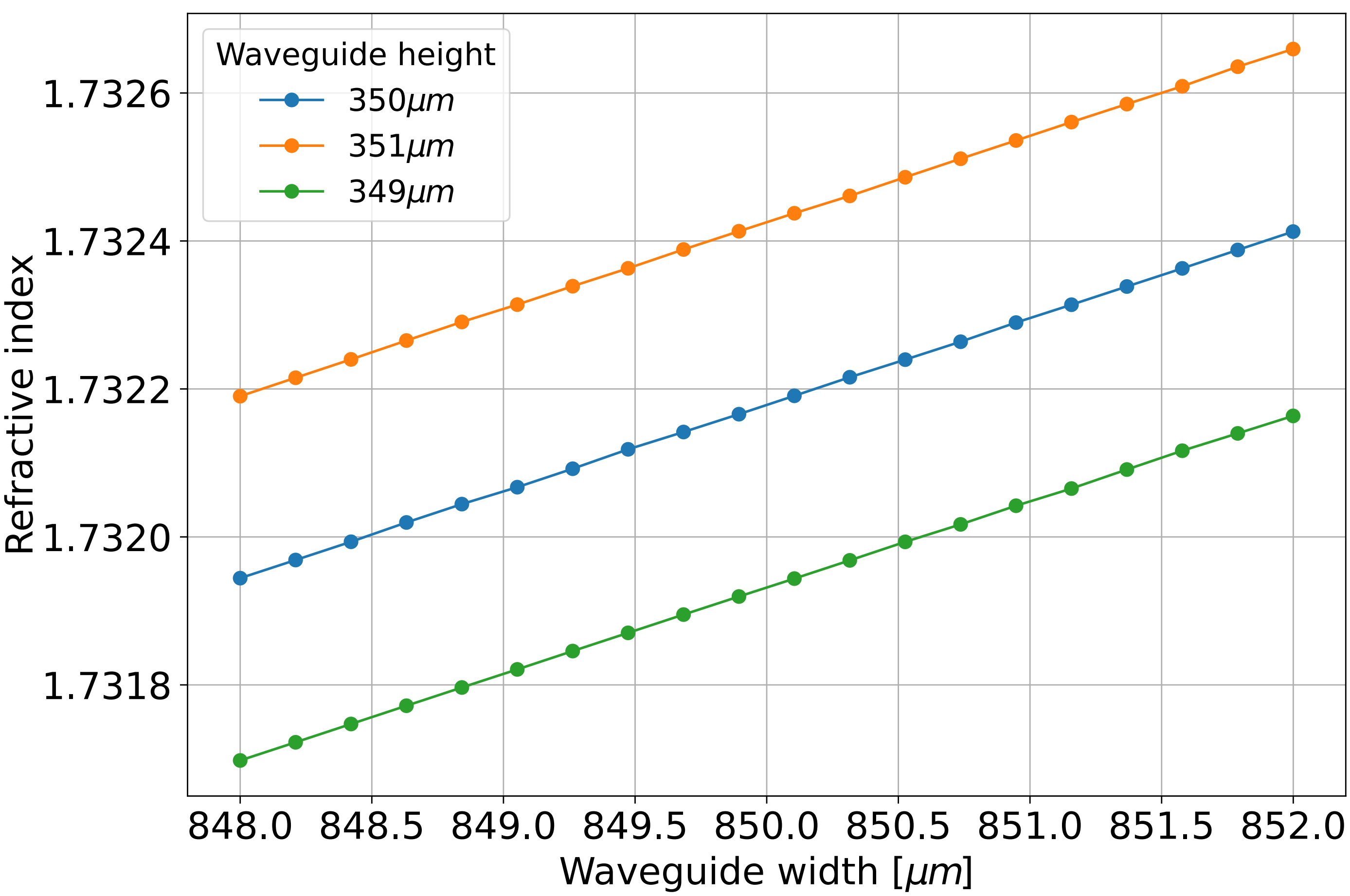

Figure 1: Simulated error on the effective refractive index, depending on different height and width of the waveguide.

estimate the error on the resonance wavelength with the formula $\Delta\lambda_{res} = \Delta n_{eff} L/m$. Here m is estimated to be between 1600 and 1800 based on the transmission spectra. The resulting expected maximum error that we expect on the resonance wavelength is in the range 0.6 nm to 0.8 nm. This is slightly higher, but still in line with the spread observed in fig. 1c in the main text. The simulated values for the refractive index can be found in the supplementary information.

5 RF measurement data

The measured RF data at different bias voltages for different rings is shown in fig. 2.

6 Long term stability

One ring resonator was poled and kept at 60V for 2.5 days (60h) every 5 minutes a measurement was performed. This is in addition to the 10h measurement in the main text. This to show the behavior does not diverge even at longer periods of time. The measurement data is shown in fig. 3. The shift over 60h is only 5% of the FSR of the ring resonators.

7 hysteresis stability

The hysteresis measurement shown in the main text was repeated 5 times in order to show the reversibility and repeatability of the two linear states shown in fig. 4. It is clear that both states can be reached at least 5 times without deteriorating, demonstrating the good stability of the thin film PZT. However, it must be noted that after extensive poling of more than 5 days, the hysteresis shifts as seen in fig. 5, where the hysteresis was repeated 10 times. The exact origins of this phenomenon must be further investigated, but our initial hypothesis consists of a combination of charge and defect migrations and relaxations in the thin films. Although this limits the ability to switch between linear regions, it still allows the operation within one linear region, which was shown to suffice for the trimming operation.

References

[1] A. Yariv, P. Yeh, *Optical Waves in Crystals: Propagation and Control of Laser Radiation*, John Wiley and Sons, **1983**.

[2] E. Picavet, K. De Geest, E. Lievens, H. Rijckaert, T. Vandekerckhove, E. Solano, D. Deduytsche, L. Van Bossele, D. Van Thourhout, K. De Buysser, J. Beeckman, *ACS Applied Materials & Interfaces* **2024**, *16*, 31 41134, pMID: 39077874.

[3] D. Taillaert, F. V. Laere, M. Ayre, W. Bogaerts, D. V. Thourhout, P. Bienstman, R. Baets, *Japanese Journal of Applied Physics* **2006**, *45*, 8R 6071.

[4] D. J. Kim, J. Y. Jo, Y. S. Kim, Y. J. Chang, J. S. Lee, J.-G. Yoon, T. K. Song, T. W. Noh, *Phys. Rev. Lett.* **2005**, *95* 237602.

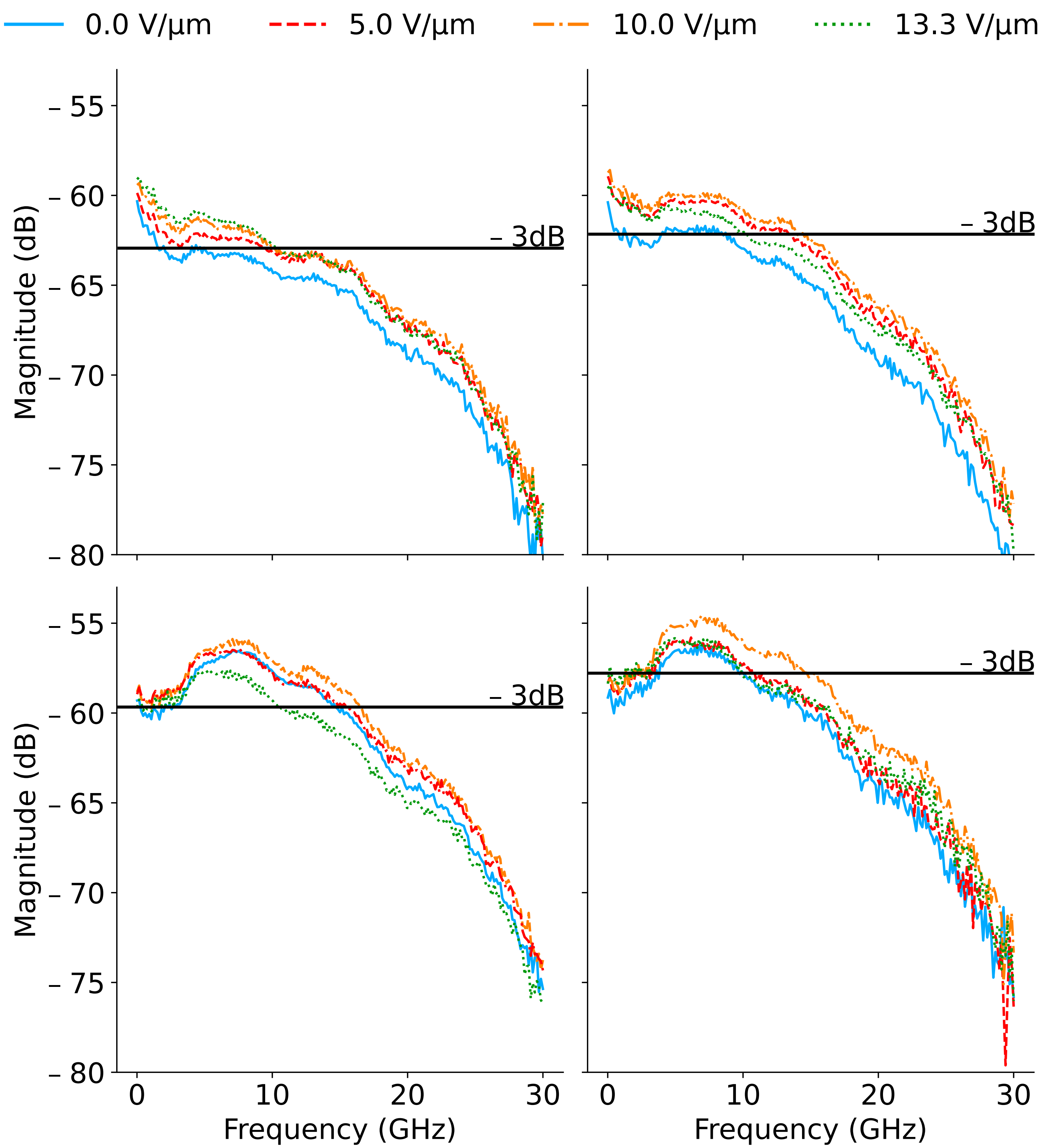

Figure 2: Measured S12 parameters of 4 rings at different applied DC bias voltages

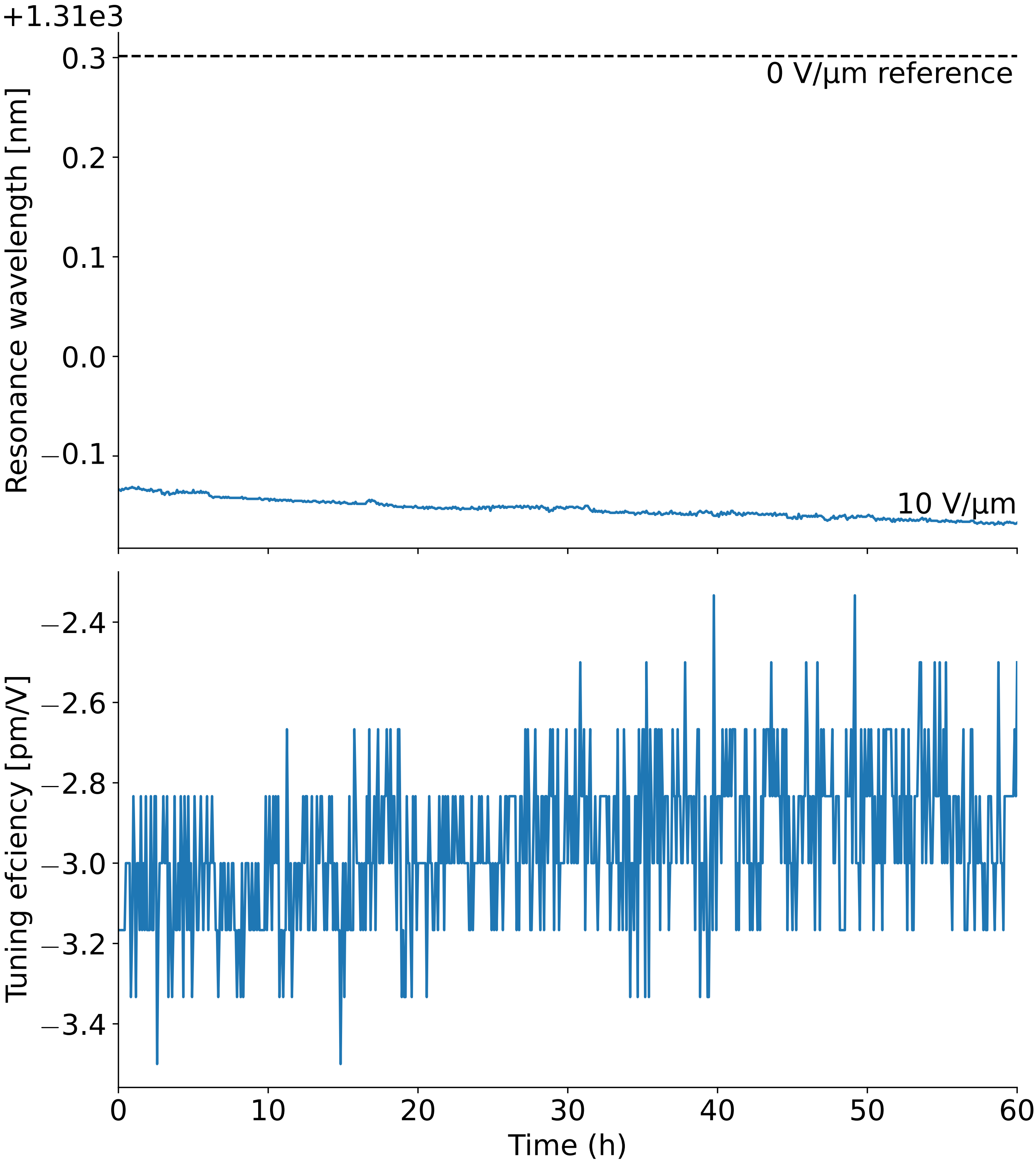

Figure 3: The resonance wavelength shift (a) and tuning efficiency (b) over time for constant DC-field of $10\,\mathrm{V\,\mu m^{-1}}$

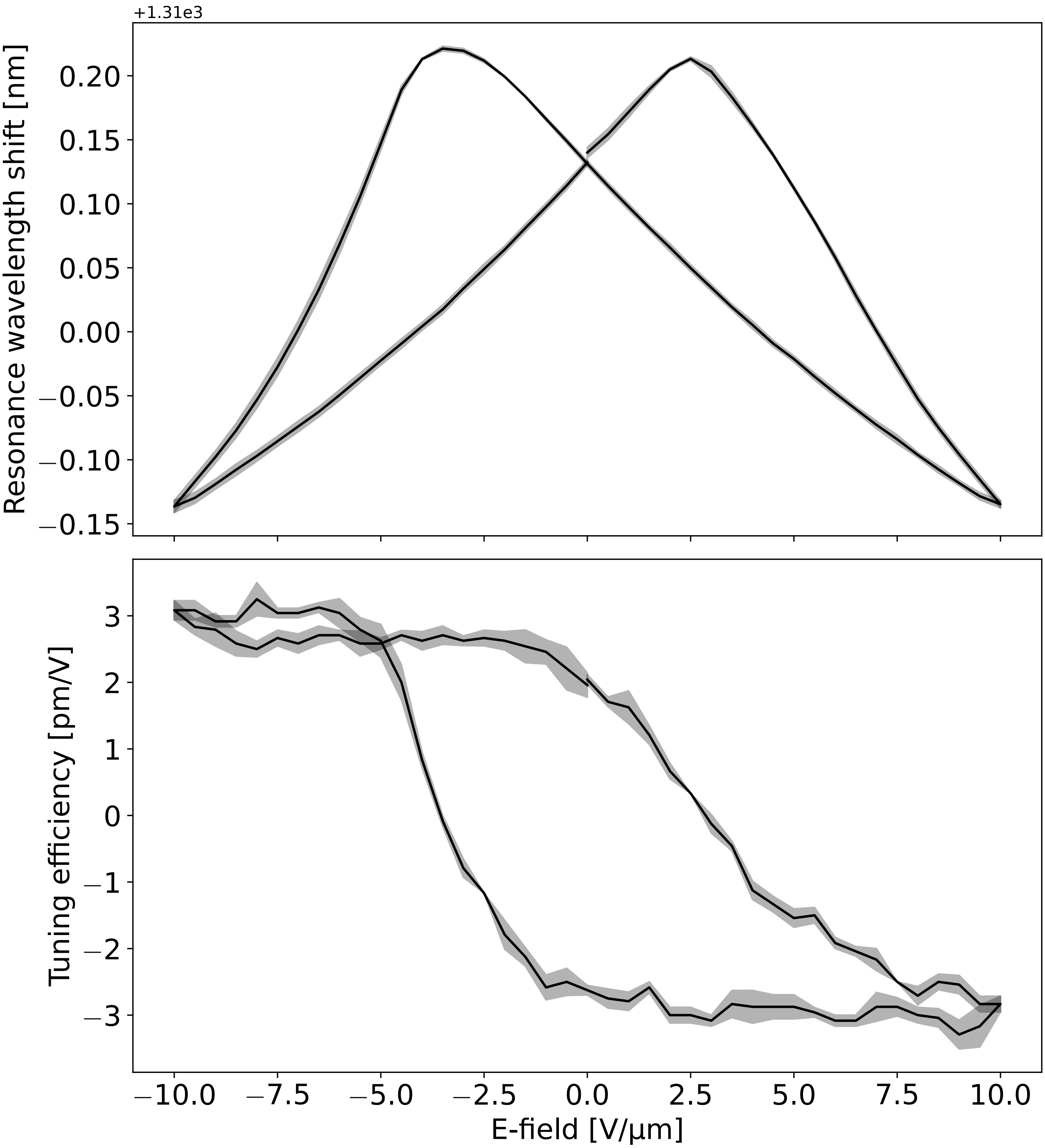

Figure 4: Five loops of the hysteresis curve of a ring resonator spanning between $\pm 10\,\mathrm{V\,\mu m^{-1}}$.

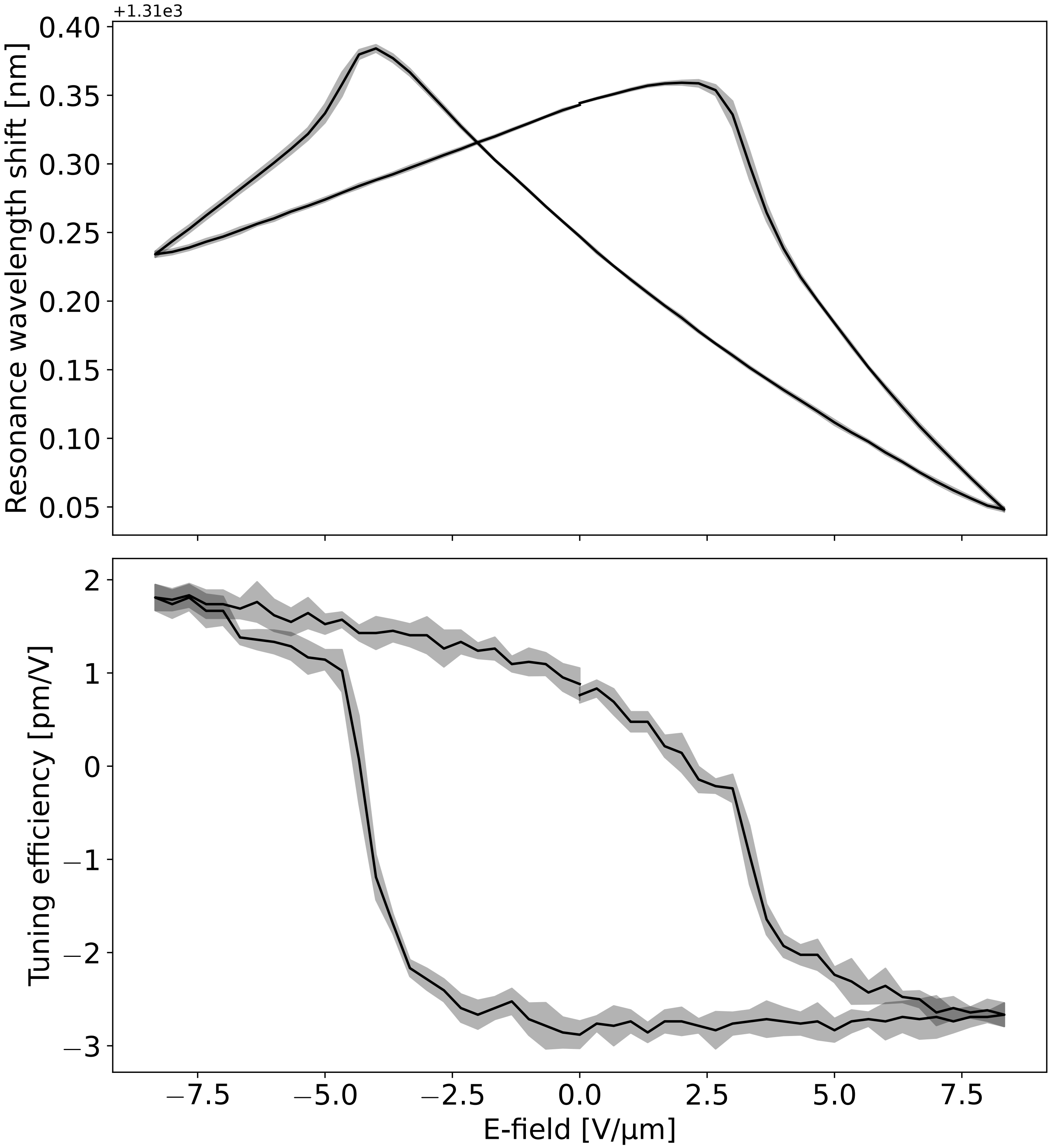

Figure 5: Ten loops of the hysteresis curve of a ring resonator spanning between $\pm 8.33\,\mathrm{V\,\mu m^{-1}}$ after it has been continuously poled for more than 5 days.
